# Urinary microbiota signatures associated with different types of urinary diversion: a comparative study

**DOI:** 10.3389/fcimb.2023.1302870

**Published:** 2024-01-03

**Authors:** Yuchao Liu, Jingcheng Zhang, Haotian Chen, Wentao Zhang, Ailiyaer Ainiwaer, Shiyu Mao, Xudong Yao, Tianyuan Xu, Yang Yan

**Affiliations:** ^1^ Department of Urology, Chongming Branch of Shanghai Tenth People’s Hospital, Tongji University, Shanghai, China; ^2^ Department of Urology, Shanghai Tenth People’s Hospital, Tongji University, Shanghai, China; ^3^ Institute of Urinary Oncology, School of Medicine, Tongji University, Shanghai, China; ^4^ Department of Urology, Kashgar Prefecture Second People Hospital, Kashgar, Xinjiang Uygur Autonomous Region, China

**Keywords:** bladder cancer, urinary diversion, microbiota, neobladder, Studer

## Abstract

**Background:**

Radical cystectomy and urinary diversion (UD) are gold standards for non-metastatic muscle-invasive bladder cancer. Orthotopic neobladder (or Studer), ileal conduit (or Bricker) and cutaneous ureterostomy (CU) are mainstream UD types. Little is known about urinary microbiological changes after UD.

**Methods:**

In this study, urine samples were collected from healthy volunteers and patients with bladder cancer who had received aforementioned UD procedures. Microbiomes of samples were analyzed using 16S ribosomal RNA gene sequencing, and microbial diversities, distributions and functions were investigated and compared across groups.

**Results:**

Highest urine microbial richness and diversity were observed in healthy controls, followed by Studer patients, especially those without hydronephrosis or residual urine, α-diversity indices of whom were remarkably higher than those of Bricker and CU groups. Studer UD type was the only independent factor favoring urine microbial diversity. The urine microflora structure of the Studer group was most similar to that of the healthy individuals while that of the CU group was least similar. Studer patients and healthy volunteers shared many similar urine microbial functions, while Bricker and CU groups exhibited opposite characteristics.

**Conclusion:**

Our study first presented urinary microbial landscapes of UD patients and demonstrated the microbiological advantage of orthotopic neobladder. Microbiota might be a potential tool for optimization of UD management.

## 1 Introduction

Bladder cancer is a common urologic malignancy, with estimates of 91,893 new cases and 42,973 deaths in China every year (Siegel et al., 2022). According to the depth of tumor invasion, it can be classified as non-muscle invasive bladder cancer (NMIBC) or muscle-invasive bladder cancer (MIBC). Considering substantial risks of metastasis and lethality, radical cystectomy (RC) and lymphadenectomy are recommended for the treatment of non-metastatic MIBC, and urinary diversion (UD) inevitably becomes the last but crucial surgical step for such cases. Digestive tracts are usually employed for UD, like stomach, ileum and sigmoid colon, and orthotopic UD (or neobladder, ONB), ileal conduit (IC) and cutaneous ureterostomy (CU) are mainstream UD procedures (Lenis et al., 2020). ONB is believed to be the gold standard for surgical bladder reconstruction following RC though it is performed less often than other UD types (Janssen et al., 2021). Studer, which are the most common orthotopic urinary diversion, provides daytime continence rate up to 92%, respectively. The main advantage of ONB is that on the basis of radical resection of bladder cancer, the normal urinary system anatomy can be approximately simulated. Since ONB procedure basically maintains a normal physiological and anatomical state, postoperative patients do not need an external stoma or carry urine collection bag. They are also likely to accept the adverse psychological stimulation brought by this surgery. Studer is the most common ONB technique which provides a daytime continence rate up to 92% (Sheybaee Moghaddam et al., 2022). As for incontinent UD procedures, Bricker is ranked as the most representative IC technique which has the advantages of shorter operation time and fewer complications compared with ONB. With the advent of robotic surgeries, IC or Briker has seen a proportionally greater increase in utilization as the number of robotic RCs being performed with intracorporeal UD rises (Janssen et al., 2021). In the long run, the advantage of Bricker over incontinent CU is that stents do not need to be replaced regularly, thus reducing the possibilities of infection and renal insufficiency as well as improving patients’ quality of life. Nonetheless, CU also shows a wide scope of application owing to relatively limited intraoperative trauma, especially for the elderly and more frail patients (Korkes et al., 2022). Shared decision-making and patient-centered approach should be used when tailoring the UD type.

As for human microorganisms, they are widely distributed in the gut, mouth, skin, bronchus, reproductive tract, organ tissues (Hou et al., 2022). In recent years, with the understanding of microorganisms, it has been found that the contribution of microorganisms to human malignant tumors is as high as 20% (Garrett, 2015).The most prominent examples are Helicobacter pylori, which is linked to stomach cancer, and the high-risk form of human papillomavirus, which is linked to cervical cancer (van Elsland and Neefjes, 2018). The interaction of microorganisms with their hosts is extremely complex, and a variety of molecular mechanisms can be envisaged through which tumor occurrence, tumor progression, and response to anticancer therapies can be influenced. Microorganisms can also induce chronic inflammation, providing a backdrop for tumor development, or trigger an immunosuppressive response that disrupts immune surveillance of cancer (Zitvogel et al., 2017). Finally, microbial metabolism of host metabolites, food components, or foreign substances may produce harmful compounds and may even promote the development of tumors in distant body parts (Roy and Trinchieri, 2017).Studies suggest that dysbiosis of microbiome has been revealed responsible for various urological disorders, such as urgent incontinence, interstitial cystitis, overactive bladder and urinary malignancies (Bhide et al., 2020; Knippel et al., 2021; Lane et al., 2022). Definitely, UD procedures will alter the original composition of urinary microbiome, which may be affected by the symbiotic collection of bacteria, fungi, parasites and viruses that inhabit the surface of our body’s epithelial barrier.

Previous or current researches of UD have been mainly focusing on surgical techniques or postoperative functional outcomes. However, urinary microbiological changes after RC with UD have not been illustrated. Here, for the first time, we aim to identify the urinary microbiota signatures for patients with different UD types and preliminarily analyze their associations with functional outcomes.

## 2 Methods

### 2.1 Study population and urine sample collection

**Figure 1:**
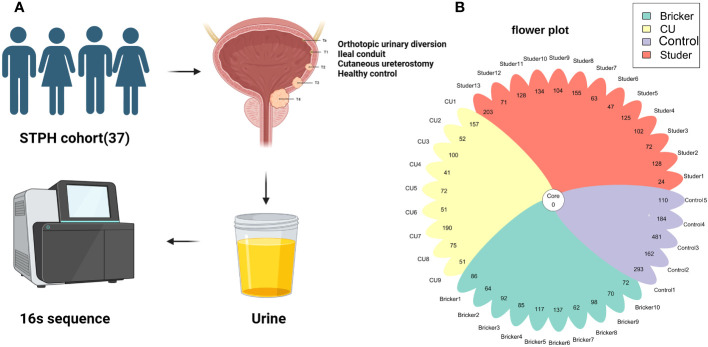
**(A)** Experimental process; **(B)** The number of amplicon sequence variants in 37 samples.

After approved by the institutional review board, patients who had received RC with UD previously and healthy controls were recruited for the current study between January and May 2023, at Shanghai Tenth People’s Hospital. Informed written consent was obtained from each participant for sample collection as well as other procedures and protocols. All UD cases had been histologically confirmed as urothelial carcinoma at RC. Those with a prior history of sexually transmitted infection were excluded. Recent urinary or systemic infection or any use of either antibiotics or probiotics (within 2 months) were also excluded. Healthy individuals volunteered as the control group, none of whom had a history of urinary infection or urologic disease. Clean-catch midstream urine samples of 50 ml from all participants were collected under the strict aseptic procedures. For IC and CU groups, urine samples were drained and collected via sterile ureteral stent and catheter, respectively. Under the guidance of professional biotechnologists, fractions for microbiota analysis from urine samples were centrifuged at 16,000 g for 10 min, and supernatants were aliquoted and stored at − 80 °C; for further processing. Meanwhile, all participants were asked to complete a structured questionnaire to gather information about socio-demographic characteristics. For UD cases, serum creatinine was detected and ultrasonography was employed to evaluate urinary tract functions (i.e., residual urine, hydronephrosis). Besides, clinicopathologic features were also extracted from the electronic medical record database (**Figure 1A**). Data collection followed the principles outlined in the Declaration of Helsinki.

### 2.2 DNA extraction and 16S ribosomal RNA (rRNA) gene amplicon sequencing

Bacterial DNA was isolated from the urine sample using a DNeasy PowerSoil kit (Qiagen, Hilden, Germany) following the manufacturer’s instructions. DNA concentration and integrity were measured by a NanoDrop 2000 spectrophotometer (Thermo Fisher Scientific, Waltham, MA, USA) and agarose gel electrophoresis, respectively. PCR amplification of the V3-V4 hypervariable regions of the bacterial 16S rRNA gene was carried out in a 25 μl reaction using universal primer pairs (343F: 5′-TACGGRAGGCAGCAG-3′; 798R: 5′-AGGGTATCTAATCCT-3′). To rule out contamination during amplification, a PCR-negative control with no template DNA was processed. The amplicon quality was visualized using gel electrophoresis. PCR products were purified with Agencourt AMPure XP beads (Beckman Coulter Inc., Brea, CA, USA) and quantified using Qubit dsDNA assay kit (Invitrogen, Life Technologies, Grand Island, NY, USA). PCR amplification was then parallel performed in 25 μl of 2 × Phanta Max Master Mix, 2 μl of forward primer (10 μM), 2 μl of reverse primer (10 μM), 50 μl of ddH2O and template DNA. The PCR was conducted under the following conditions: 95°C for 3 min; 25 cycles of 95°C for 30 s, 55°C for 30 s, and 72°C for 30 s; and a final extension at 72°C for 5 min. The concentrations were then adjusted for sequencing, which was performed on an Illumina NovaSeq6000 with two paired-end read cycles of 250 bases each (Illumina Inc., San Diego, CA, USA; OE Biotech Co., Ltd., Shanghai, China).

### 2.3 Bioinformatic analysis

Raw sequencing data were in FASTQ format. Paired-end reads were preprocessed using cutadapt software to detect and cut off the adapter. After trimming, paired-end reads were filtered low quality sequences, denoised, merged and detect and cut off the chimera reads using DADA2 (Callahan et al., 2016) with the default parameters of QIIME2 (Bolyen et al., 2019) (2020.11). At last, the software exported the representative reads and the amplicon sequence variant (ASV) abundance table. The representative read of each ASV was selected using QIIME2 package. All representative reads were annotated and blasted against Silva database Version 138 (or Unite) (16s/18s/ITS rDNA) using q2-feature-classifier with the default parameters. The microbial diversity in urine content samples was estimated using the α-diversity indices, including abundance-based coverage estimator (ACE), phylogenetic diversity whole tree, Chao1, Shannon (Chao and Bunge, 2002), observed species and Simpson (Hill et al., 2003). The Unifrac distance matrix performed by QIIME software was used for unweighted Unifrac Principal coordinate analysis (PCoA) and phylogenetic tree construction.

### 2.4 Statistical analysis

Data are expressed as mean ± standard deviation (SD). Statistical comparisons of continuous variables describing clinical features were made using the Student’s t-test, where the data were normally distributed. Chi-square test was used for categorical variables. For bacterial abundance analysis, the data were first normalized and univariate analysis of variance or Kruskal-Wallis test was used for the significant difference in relative abundance of genera. The differences of metabolite abundance in Studer group, Bricker group and CU group were analyzed by student t-test. Logistic regression was used to analyze the associations between clinicopathologic factors and urine microbial diversities. P <0.05 was considered statistically significant, and the P value was corrected by Benjamini-Hochberg FDR.

## 3 Results

### 3.1 Subject characteristics

In this study, 16S rRNA gene sequencing was performed on qualified clean midstream urine samples from 5 healthy volunteers and 32 patients who had received RCs and UDs, including 13 Studers, 10 Brickers and 9 CUs. The clinical and demographic characteristics of four groups as well as oncologic features of UD cases are shown in **Table 1**. There was no statistically significant difference in age, gender, BMI or metabolic comorbidities among these four groups. In terms of UD groups, there was no significant difference in either pathologic T stage at RC or proportion of recurrence. The mean duration from RC to urine sample collection was 48 months for Studer patients, which was significantly longer than other two UD groups (Bricker: 40, CU: 40, P = 0.052).

**Table 1:** Clinicopathologic characteristics of each group.

Variables	Studer n =13	Bricker n = 10	CU n = 9	Control n = 5	P-value
Age (y)	67.5(64.0, 74.0)	71.9(64.0, 79.0)	71. 8(60.0, 85.0)	70.8(63.0, 76.0)	0.2355
Gender					0.1051
male	13	8	6	5	
female	0	2	3	0	
BMI	23.9(17.3, 29.8)	22.7(17.8, 26.6)	23.6(18.3, 26.0)	23.9(21.9, 25.3)	0.7845
Hypertension					0.7640
Yes	5	5	5	1	
No	8	5	4	4	
Diabetes					0.7103
Yes	3	4	2	1	
No	10	6	7	4	
T stage					0.2869
T1	4	4	1	\	
T2	7	5	4	\	
T3-4	2	1	4	\	
UD duration (month)	48 (12,120)	40(12,120)	40(12,96)	\	0.0522
Recurrence					0.3046
Yes	3	3	4		
No	10	7	5		

Data are presented as average (minimum value, maximum value) for continuous variables or n (%) for counts; BMI, body mass index; n, number of people; \ , not applicable.

### 3.2 Urine microbial diversities of healthy controls and UD individuals

**Figure 2:**
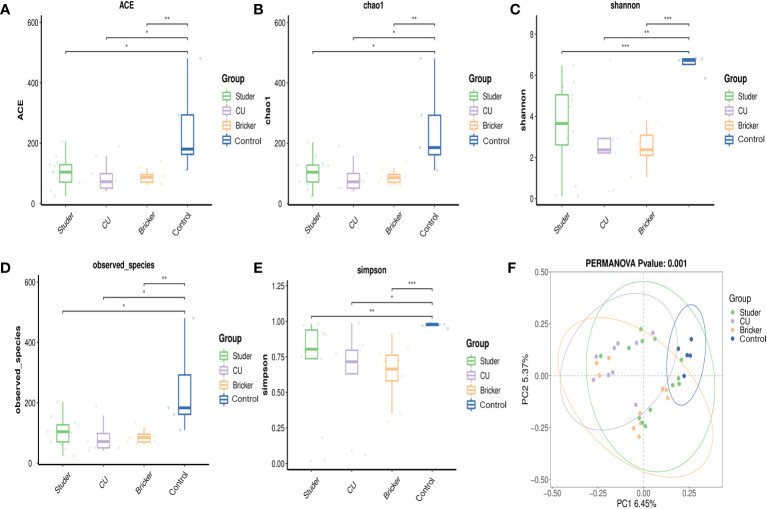
α- and β-diversity indices for Studer, Bricker, cutaneous ureterostomy samples and healthy control samples. **(A)** abundance-based coverage estimator index; **(B)** Chao1 index; **(C)** Shannon index; **(D)** observed species index; **(E)** Simpson index; **(F)** principal coordinate analysis based on Adonis test (a dot representing each sample; F = 2.53, P < 0.001). * P <0.05, ** P <0.01, *** P <0.001.

Illumina sequencing of the V3-V4 hypervariable region of 16S rRNA amplicons from all samples yielded 78108 raw reads and 81970 reads after pre-processing (**Figure 1A**). The sequence counts per sample ranged from 24 to 481 reads. In total, 2543 ASVs were detected and the mean ASVs of urine samples varied among groups, which were 104, 88, 88 and 246 for Studer, Bricker, CU subjects and healthy controls, respectively (**Figure 1B**). The ASV data volume and categorical information indicated significant differences among groups (**Table S1**). We evaluated microbial diversities of urine samples, and there was no statistically significant difference in ACE, Chao1, Shannon, observed species or Simpson index among four groups. However, remarkably higher values of these α-diversity indices were unanimously observed in the control group, followed by Studer patients, who were closer to healthy volunteers than counterparts of other UD types (**Figure 2**). Furthermore, we found that Studer UD type was the only independent clinicopathologic factor favoring urine microbial diversity of UD patients in multivariate regression analyses for both Shannon and Chao1 indices (**Tables 2**, **S2**). Interestingly, we also analyzed the associations between urine microbial diversity and Studer ONB functions. As shown in **Supplementary Figure S1**, urine samples from Studer subjects without hydronephrosis or residual urine showed higher α-diversity indices, which were closer to samples from healthy controls.

**Table 2:** Logistic regression analysis on α-diversity (Shannon index) for urinary diversion samples.

α-diversity (Shannon index)	B	P	OR	95% CI	
				Upper limit	Lower limit
UD type (Studer vs non-Studer)	-2.627	0.024	0.072	0.007	0.711
Age (y)	-0.136	0.928	0.873	0.046	16.584
Sex (male vs female)	-0.035	0.826	0.966	0.707	1.319
BMI	-0.322	0.806	0.725	0.056	9.447
Recurrence (yes vs no)	-1.091	0.358	0.336	0.033	3.439
Pathological stage (≤T2 vs >T2)	0.044	0.666	1.045	0.855	1.278
UD duration (y)	-0.005	0.792	0.995	0.961	1.31

*The first third of Shannon indices was adopted as cutoff value for regression analysis.

In this study, PCoA was also used to compare the similarity in the microbial community composition of urine specimens. As shown in **Figure 2F**, there was no obvious difference in the distribution of bacteria when comparing Studer group with healthy individuals or Bricker group, but a relatively remarkable difference between Bricker group and healthy controls. As for CU subjects, their urine bacteria distribution was significantly different from those of both Studer and Bricker counterparts, let alone the control group. Overall, the PERMANOV test showed that the observed difference was statistically significant (Adonis test, Binary-Jaccard, F=1.4795, P<0.001). Similarly, PCoA based on ONB functions in the Studer cohort also revealed significant results of β-diversity that microbial community composition of urine samples from those without hydronephrosis or residual urine was more similar to the control group (**Supplementary Figures S1E, F**).

### 3.3 Urine microbial community distributions of healthy controls and UD individuals

**Figure 3:**
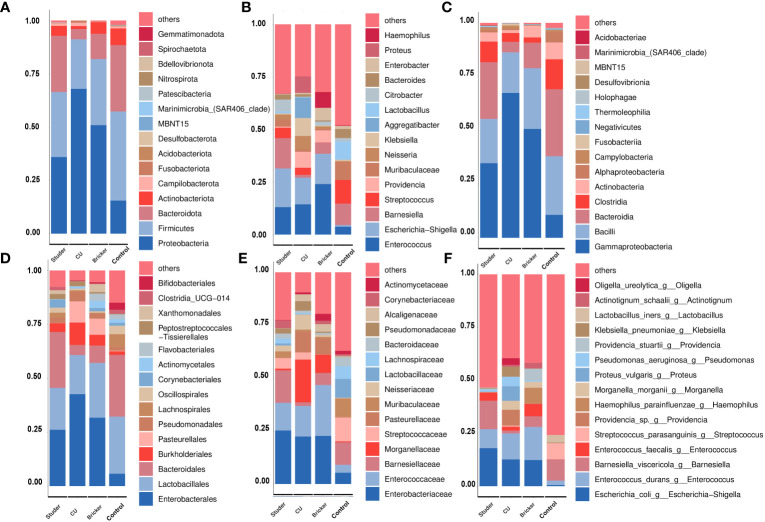
Average relative abundances of major taxa represented by bar graphs. **(A)** phylum; **(B)** genus; **(C)** class; **(D)** order; **(E)** family; **(F)** species. Each colored box represents a bacterial taxon and the height of a colored box represents the relative abundance of the organism within the urine samples. Bacterial genera with a relative abundance <1% or those unclassified are grouped as “Others”.

To explore the microbial signature alteration after UD, we evaluated the relative abundance of taxa in different groups. At the phylum level, the majority of dominant bacteria were from Proteobacteria (Studer: 35.9%, Bricker: 50.9%, 67.9% CU: 67.9%, control: 15.5%), Firmicutes (Studer: 30.5%, Bricker: 31.0%, CU: 23.2%, control: 41.8%) and Bacteroidota (Studer: 26.4%, Bricker: 11.9%, CU: 4.8%, control: 31.2%; **Figure 3A**). At the generic level, three dominants were Enterococcus (Studer: 13.1%, Bricker 14.4%, CU: 24.0%, control: 3.7%), Escherichia-Shigella (Studer: 18.4%,12.7% Bricker: 12.7%, CU: 14.4%, control: 0.1%) and Barnesiella (Studer 14.4%, Bricker: 5.3%, CU: 1.3%, control: 10.2%; **Figure 3B**). It is worth noting that the Escherichia-Shigella genus was frequently detected in three UD groups, but rarely in the control group. By contrast, Barnesiella was the second most dominant genus in urine samples from both healthy volunteers and Studer subjects, far more often than in samples from Bricker and CU counterparts. Urine microbial communities of different groups at class, order, family and species levels are shown in **Figures 3C–F**, respectively. Overall, the main urine microflora structures were comparable among different UD types, whereas Studer and CU groups showed the most and least similar to healthy controls, respectively.

**Figure 4:**
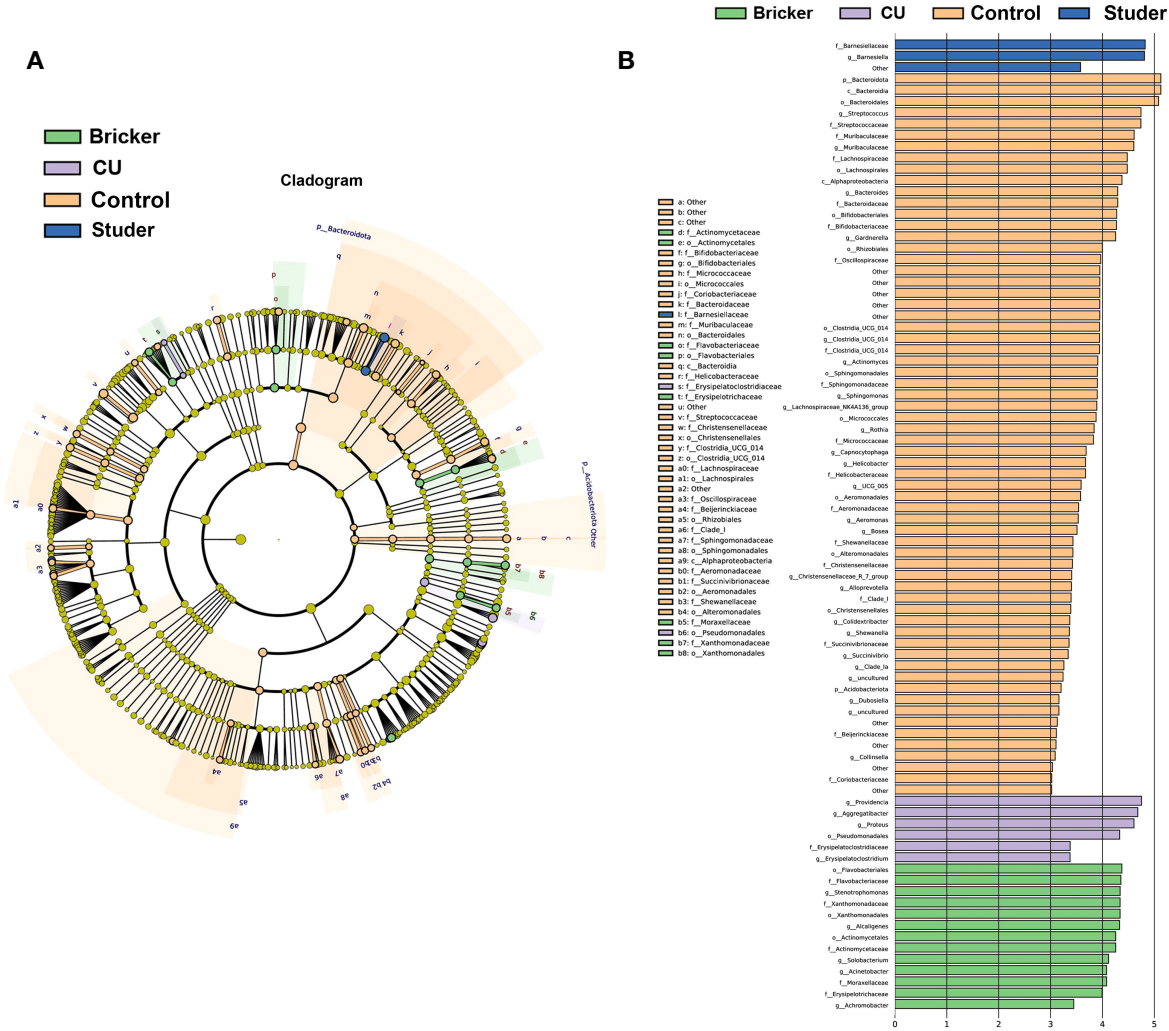
Microbial taxa associated with urine samples from patients of urinary diversion and control subjects. **(A)** cladogram representation of the urine microbial taxa associated with urinary diversion and control samples; **(B)** association of specific urine microbiota taxa with different groups by linear discriminant analysis effect size.

To identify specific taxa associated with UD types, linear discriminant analysis effect size (LEfSe) was further performed. As shown in **Figure 4**, Actinomycetaceae, Solobacterium, Stenotrophomonas, Xanthomonadales and Flavobacteriales were significantly enriched in Bricker group. Abundances of Aggregatibacter, Proteus, Providencia and Erysipelatoclostridiaceae were relatively increased in CU group. Healthy controls were characterized by enrichment of Streptococcus, Muribaculaceae, Gardnerella and Bacteroides, at the generic level. In Studer group, the only abundant genus, Barnesiella, belongs to the family Porphyromonadaceae, within the phylum Bacteroidetes, which was also the most abundant taxa in the control group.

### 3.4 Microbial functional alterations associated with UD types

**Figure 5:**
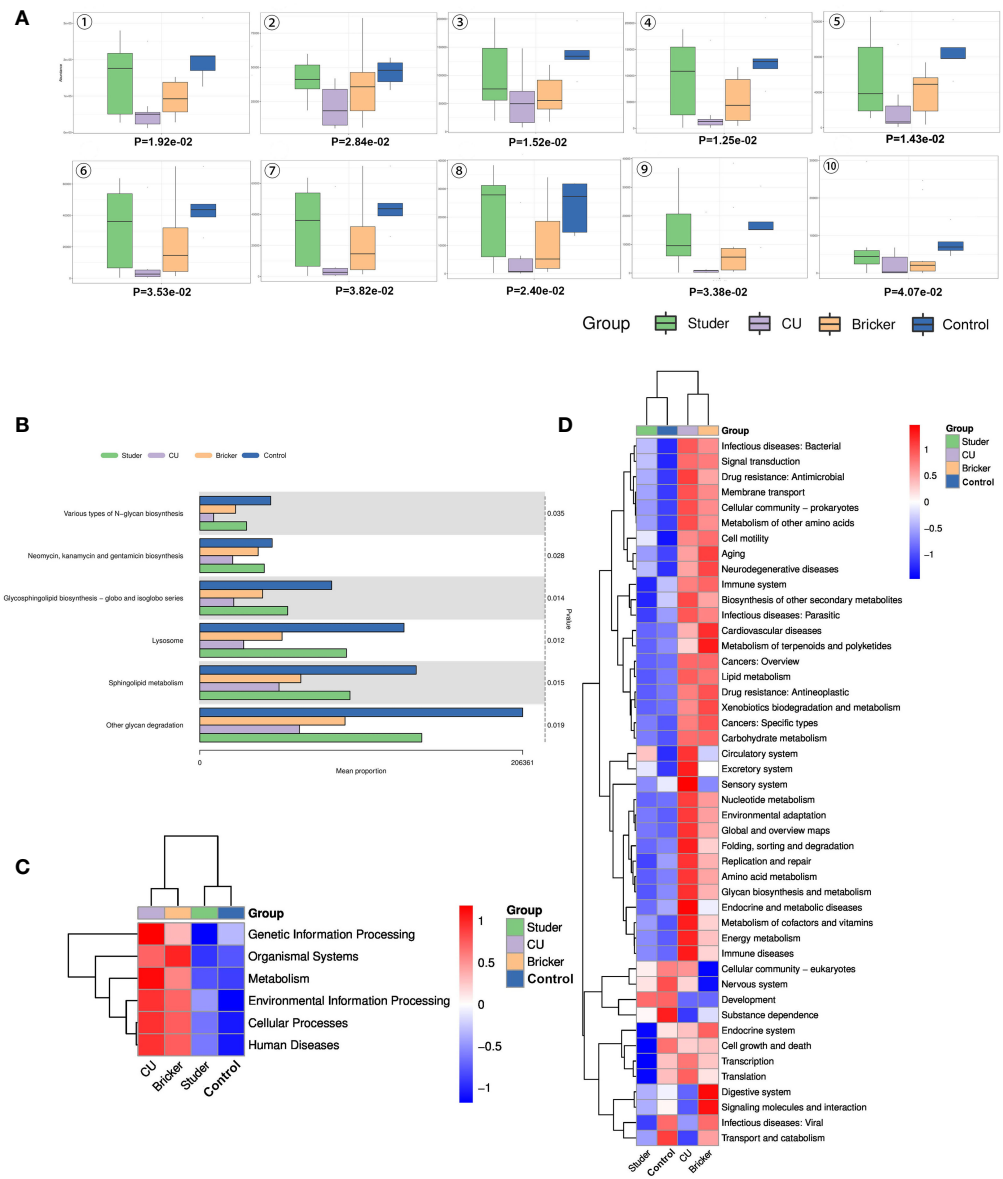
Differential KEGG functions among groups. **(A)** box plots of differential KEGG functions: 1. other glycan degradation, 2. neomycin, kanamycin & gentamicin biosynthesis, 3. sphingolipid metabolism, 4. lysosome, 5. glycosphingolipid biosynthesis - globo & isoglobo series, 6. various types of N-glycan biosynthesis, 7. glycosphingolipid biosynthesis - ganglio series, 8. N-glycan biosynthesis, 9. protein digestion & absorption, 10. meiosis - yeast; **(B)** bar charts of top differential KEGG functions; **(C, D)** level 1 and 2 clustering heat maps of KEGG results.

To infer functional pathways from microbial community profiles, phylogenetic investigation of communities by reconstruction of unobserved states (PICRUSt) was used, and microbial functional differences between UD patients and healthy controls were investigated using the Kyoto encyclopedia of genes and genomes (KEGG) analysis. In this way, Studer, Bricker, CU and control groups can be clearly distinguished, as shown by **Figure 5A**. Microbial genes predicted to be significantly enriched in Studer group touched on glycan degradation, N-glycan biosynthesis, sphingolipid metabolism, lysosome, glycosphingolipid biosynthesis as well as neomycin, kanamycin and gentamicin biosynthesis (**Figure 5B**). Importantly, based on the clustered heat map of KEGG analysis, Studer patients and healthy volunteers shared similar urine microbial gene functions at different level. (**Figures 5C, D**). For example, Studer and control groups were both significantly enriched in microbial genes associated with development, which were downregulated in Bricker and CU groups. By contrast, negatively functional genes associated with infectious diseases and drug resistance were lowly expressed in Studer and control groups, while both enriched in Bricker and CU groups (**Figure 5D**).

**Figure 6:**
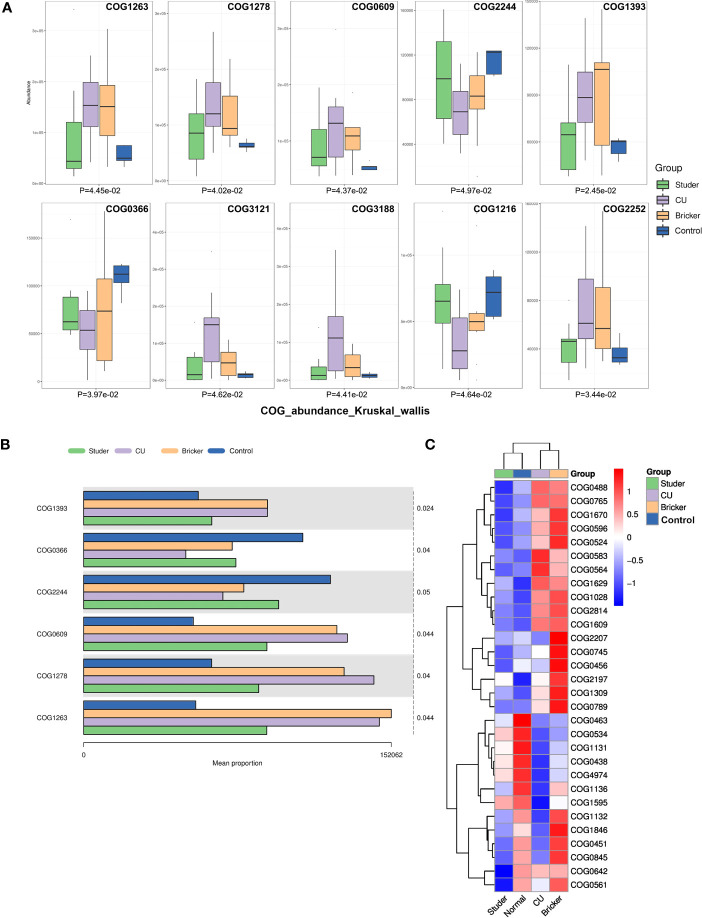
Differential clusters of orthologous groups of proteins among groups. **(A, B)** box plots and bar charts of top differential clusters of orthologous groups of proteins; **(C)** clustering heat map of differential clusters of orthologous groups of proteins.

In addition, we functionally annotated the genes by searching the clusters of orthologous groups of proteins (COG) database. The first 10 differential COGs were shown, as well as the average abundance of the first 6 differential COGs in each group. Based on the clustered heat map of the COG difference results, we also found Studer patients and healthy volunteers shared many similar urine microbial genes, which were inversely expressed in Bricker and CU groups (**Figures 6A–C**).

## 4 Discussion

Reduced microbial diversity has been recognized as a feature in intestinal diseases such as ulcerative colitis, Crohn’s disease and colorectal cancer (Lepage et al., 2011; Ahn et al., 2013; Gevers et al., 2014). However, such consistent microbial finding was not confirmed in urinary disorders. Increased microbial diversity was observed in urge incontinence and decreased in interstitial cystitis and overactive bladder, while no significant difference was found in bladder cancer (Zhang et al., 2023). To date, the urine microbial community of patients undergoing UD for bladder cancer has not been adequately studied in comparison to the urine microbiota signatures of bladder cancer. Here, we performed 16s rRNA and bioinformatic analyses to comprehensively characterize the urinary microbial landscapes and potential functional impacts among UD patients. Overall, there were no significant differences among the three groups of UD patients regarding urine microbial diversities, all of which showed remarkably less levels than healthy individuals. However, Studer or ONB surgery, was found to be a factor favoring urine microbial diversity among these patients, showing urinary microbiota signatures most approximate to the control group. In PCoA analysis, Studer group and healthy individuals showed most similar distribution of urine bacteria, whereas CU subjects manifested with totally different urine microbial characteristics. It can be speculated that UD type should be a key factor determining individual urine microbiota. Incontinent UD approaches with abdominal stoma are more liable to affected by exotic microorganism, and this was exacerbated by long-term ureteral stenting among CU patients. ONB procedure would not only mimick the enclosed physiological alignment but also render the microbial environment closest to the original urinary system, thus providing more favorable functional outcomes.

Proteobacteria are considered to be intestinal symbiotic bacteria with pathogenic capacity (Puri, 2019). Increases in intestinal proteobacteria have been reported in colitis-associated colorectal cancer (Quaglio et al., 2022). Proteobacteria may also play an important role in the development of bladder cancer (Liu et al., 2019). Thus, the elevation of Proteobacteria may be considered as unfavorable ecological dissonance, and it was more obvious in Bricker and CU groups, which were less similar to the original alignment than Studer approach. Firmicutes and Bacteroidetes are healthy indicators which vary with age, diet quality and environmental factors. Low flora ratios may be associated with obesity, inflammatory bowel disease, cardiovascular disease and colorectal cancer (Sun and Kato, 2016; Singh et al., 2017; Klement and Pazienza, 2019). Our analysis showed that Firmicutes and Bacteroidetes accounted for 73% of urine bacteria in healthy individuals, 57% in the Studer group, 43% in the Bricker group and 28% in the CU group. Distributions of these major probiotics also indicate that ONB has a clear microbial advantage as the procedure closest to the natural cavity. By contrast, Escherichia-Shigella is one of pathogens that cause intestinal infection (Yacouba et al., 2022), while conflicting results regarding urine Escherichia-Shigella have been reported in patients with bladder cancer (Liu et al., 2019; Hussein et al., 2021). The abundance of Escherichia-Shigella was higher in the Studer group than the other groups. Escherichia-Shigella infection of macrophages and epithelial cells induces a strong inflammatory response and macrophage death (Kotloff, 2022). The definite role of Escherichia Shigella should be further evaluated in UD patients.

This study was based on genera changes to characterize urine ecological disorders, and UD cohorts and healthy controls could be perfectly separated. Within the current sample size, no statistically significant difference of α or β-diversity was observed among sub-cohorts grouped by gender, age, BMI, initial tumor stage, current creatinine or post-UD duration. Interestingly, after dichotomizing the Studer subjects based on urinary functions, those without hydronephrosis or residual urine were closer to the healthy individuals in these microbial indices, implying the potential link of urine microbiota with UD functions. Further studies should be carried out to validate our preliminary finding as well as illustrate related microbial mechanisms.

In our study, LEfSe analysis showed that bacterial groups along the Barnesiellaceae to Barnesiella lineage were enriched in Studer patients. Specific function of such flora remains to be explored. In Bricke and CU groups, there were obvious increases in pathogenic bacteria communities, which resulted in higher possibilities of postoperative urinary infections and suboptimal functional outcomes. Solobacterium is considered to be an opportunistic pathogen and important member of oral microorganisms, mainly causing oral diseases (Barrak et al., 2020). Staphylococcus moskii has been reported to cause a variety of infections, such as blood and surgical wound infections (Zheng et al., 2010; Vancauwenberghe et al., 2013). In the Bricker group, high levels of such flora may suggest an increased risk of postoperative infection. Proteus, a member of the Enterobacteriaceae family, is generally considered a low-abundance symbiotic bacteria in the gut and most commonly cited clinically as the cause of urinary infections (Hamilton et al., 2018). In the CU group, we found a significant increase of Proteus, suggesting inevitably higher risk of infections. Overall, these microflora in urine may be a potential biomarker or tool to optimize UD management.

Our study has some limitations. First was the relatively small sample size. Due to the fact that Studer surgery is more commonly performed in men, only male ONB patients were included in this study. This study was also limited to retrospective design that could not determine the causal relationship between the microbiome and post-UD recovery. Hence, prospective studies in larger cohorts are needed. In addition, the ONB or IC mucosa would be better to characterize the microbiota signatures and rule out contamination by confounding microorganisms. Fundamental researches may help elucidate the role of microbiome in different stages after UD.

## 5 Conclusion

For the first time, we presented comprehensive urine microbial landscapes of UD patients and preliminarily revealed the association of urine microbial features with function outcomes in the current study. Among UD cases, Studer type was the only independent clinicopathologic factor favoring urine microbial diversity, and Studer patients, especially those with perfect ONB functions, exhibit urinary microbiota signatures most approximate to healthy people. Further studies should be carried out to illustrate microbial mechanisms related with the regulation of UD functions.

## Data availability statement

The data presented in the study are deposited in the NCBI-Sequence Read Archive (SRA), accession number PRJNA1048651.

## Ethics statement

The studies involving human participants were reviewed and approved by the Ethics Committee of Shanghai Tenth People’s Hospital. The patients/participants provided their written informed consent to participate in this study.

## Author contributions

YL: Writing – original draft. JZ: Data curation, Writing – review & editing. HC: Writing – review & editing. WZ: Writing – review & editing. AA: Writing – review & editing. SM: Writing – review & editing. XY: Writing – review & editing. TX: Writing – review & editing. YY: Conceptualization, Investigation, Software, Writing – review & editing.

## Supplementary material

Supplementary Figure 1 α- and β-diversity indices for control and Studer samples. **(A-C)** α- and β-diversity indices for control samples, Studer samples with or without hydronephrosis; **(D-F)** α- and β-diversity indices for control samples, Studer samples with or without residual urine.
