# Supplementary figures and images for: Urinary microbiota signatures associated with different types of urinary diversion: a comparative study
Source: Front Cell Infect Microbiol. 2024 Jan 3;13:1302870. doi: 10.3389/fcimb.2023.1302870 (PMC10791864; doi:10.3389/fcimb.2023.1302870)

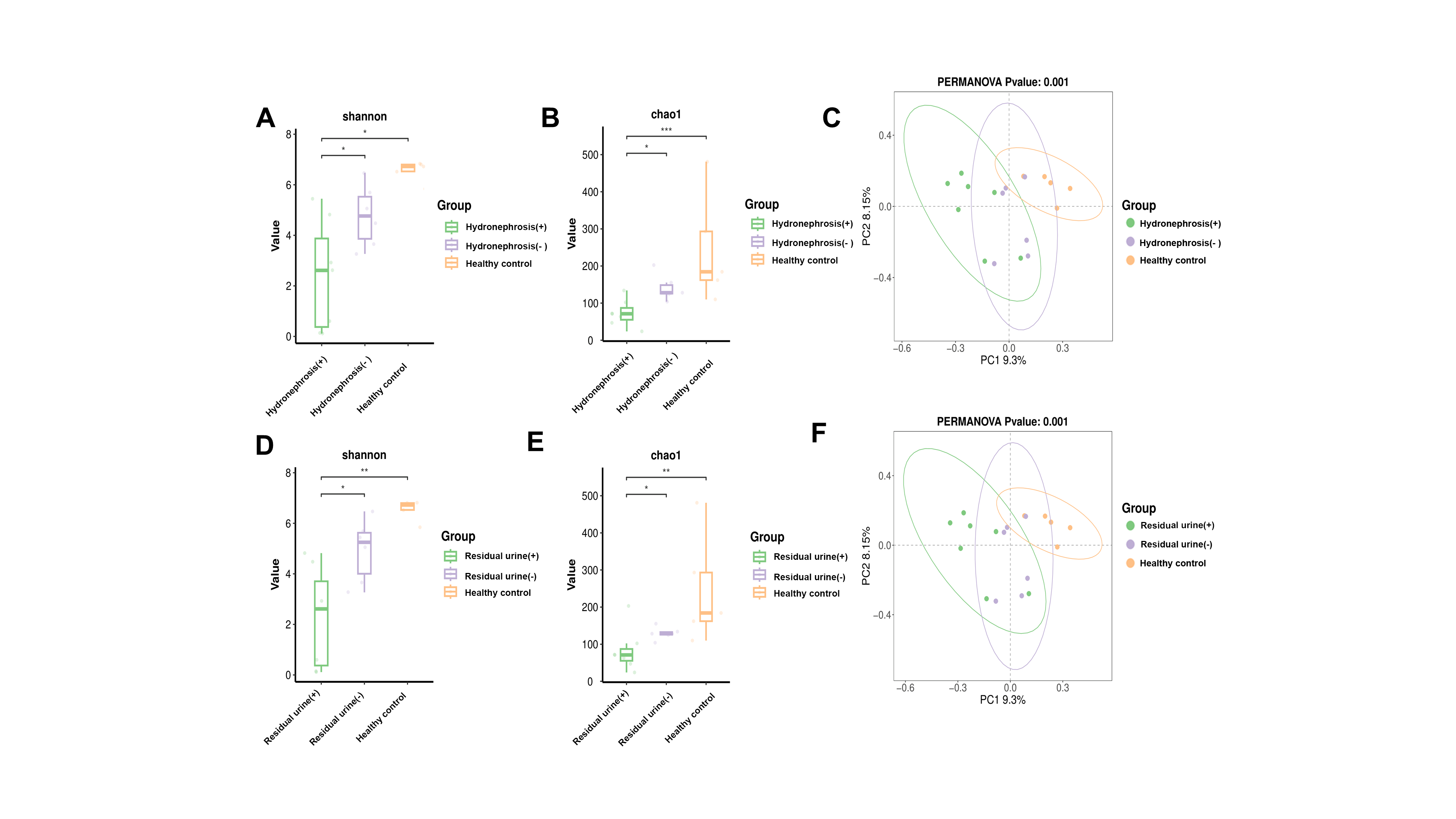

Supplement: Supplementary Figure 1 — α- and β-diversity indices for control and Studer samples. (A-C) α- and β-diversity indices for control samples, Studer samples with or without hydronephrosis; (D-F) α- and β-diversity indices for control samples, Studer samples with or without residual urine. [file Image_1.tif]
