# Supplementary material for: Urinary microbiota signatures associated with different types of urinary diversion: a comparative study
Source: Front Cell Infect Microbiol. 2024 Jan 3;13:1302870. doi: 10.3389/fcimb.2023.1302870 (PMC10791864; doi:10.3389/fcimb.2023.1302870)
Supplement: Supplementary file 3 [file Table_2.docx]

**Supplementary Table S2**| Logistic regression analysis on α-diversity (chao1 index) for UD samples

| α-diversity (chao1 index) | B | P | OR | 95% CI | |
| --- | --- | --- | --- | --- | --- |
|  |  |  |  | Upper limit | Lower limit |
| UD type  (Studer vs non-Studer) | -2.501 | 0.040 | 0.082 | 0.008 | 0.893 |
| Age (y) | -0.737 | 0.610 | 0.479 | 0.028 | 8.095 |
| Sex (male vs female) | 0.070 | 0.666 | 1.073 | 0.780 | 1.474 |
| BMI | -0.584 | 0.668 | 0.558 | 0.039 | 8.049 |
| Recurrence (yes vs no) | -1.425 | 0.252 | 0.241 | 0.021 | 2.756 |
| Pathological stage (≤T2 vs >T2) | 0.117 | 0.254 | 1.124 | 0.920 | 1.373 |
| UD duration (y) | -0.018 | 0.382 | 0.982 | 0.943 | 1.023 |

*The first third of chao1 indices was adopted as cutoff value for regression analysis.
